# Supplementary material for: Resolving the full spectrum of human genome variation using Linked-Reads
Source: Genome Res. 2019 Apr;29(4):635–45. doi: 10.1101/gr.234443.118 (PMC6442396; doi:10.1101/gr.234443.118)
Supplement: Supplemental Material [file supp_gr.234443.118_Supplemental_longranger-2.2.zip › longranger-2.2.tar.bz2!/lr2.2-public/lib/stan/cmdstan/stan/doc/api/html/math_2error__handling_2matrix_2check__pos__definite_8hpp_source.html]

Stan: src/stan/math/error\_handling/matrix/check\_pos\_definite.hpp Source File


|  |  |
| --- | --- |
|  | Stan  2.5.0  probability, sampling & optimization |


- Main Page
- Related Pages
- Namespaces
- Classes
- Files

- File List
- File Members

All Classes Namespaces Files Functions Variables Typedefs Enumerations Enumerator Friends Macros Pages

- src
- stan
- math
- error\_handling
- matrix

check\_pos\_definite.hpp

Go to the documentation of this file.

1 #ifndef STAN\_\_MATH\_\_ERROR\_HANDLING\_\_MATRIX\_\_CHECK\_POS\_DEFINITE\_HPP

2 #define STAN\_\_MATH\_\_ERROR\_HANDLING\_\_MATRIX\_\_CHECK\_POS\_DEFINITE\_HPP

3

4 #include <sstream>

5 #include <stan/math/matrix/Eigen.hpp>

6 #include <stan/math/error\_handling/dom\_err.hpp>

7 #include <stan/math/error\_handling/check\_not\_nan.hpp>

8 #include <stan/math/error\_handling/matrix/constraint\_tolerance.hpp>

9

10 namespace stan {

11

12  namespace math {

13

27  // FIXME: update warnings (message has (0,0) item)

28  template <typename T\_y, typename T\_result>

29  inline bool check\_pos\_definite(const char\* function,

30  const Eigen::Matrix<T\_y,Eigen::Dynamic,Eigen::Dynamic>& y,

31  const char\* name,

32  T\_result\* result) {

33  if (y.rows() == 1 && !(y(0,0) > CONSTRAINT\_TOLERANCE)) {

34  std::ostringstream message;

35  message << name << " is not positive definite. "

36  << name << "(0,0) is %1%.";

37  std::string msg(message.str());

38  return dom\_err(function,y(0,0),name,msg.c\_str(),"",result);

39  }

40  for (int i = 0; i < y.size(); ++i)

41  check\_not\_nan(function, y(i), "", result);

42

43  Eigen::LDLT< Eigen::Matrix<T\_y,Eigen::Dynamic,Eigen::Dynamic> > cholesky

44  = y.ldlt();

45  if (cholesky.info() != Eigen::Success

46  || !cholesky.isPositive()

47  || (cholesky.vectorD().array() <= CONSTRAINT\_TOLERANCE).any()) {

48  std::ostringstream message;

49  message << name << " is not positive definite. "

50  << name << "(0,0) is %1%.";

51  std::string msg(message.str());

52  return dom\_err(function,y(0,0),name,msg.c\_str(),"",result);

53  }

54  return true;

55  }

56

57  }

58 }

59 #endif

check\_not\_nan.hpp

dom\_err.hpp

stan::math::dom\_err

bool dom\_err(const char \*function, const T &y, const char \*name, const char \*error\_msg, const T\_msg error\_msg2, T\_result \*result)

**Definition:** dom\_err.hpp:36

constraint\_tolerance.hpp

stan::math::CONSTRAINT\_TOLERANCE

const double CONSTRAINT\_TOLERANCE

The tolerance for checking arithmetic bounds In rank and in simplexes.

**Definition:** constraint\_tolerance.hpp:11

stan::math::check\_pos\_definite

bool check\_pos\_definite(const char \*function, const Eigen::Matrix< T\_y, Eigen::Dynamic, Eigen::Dynamic > &y, const char \*name, T\_result \*result)

Return true if the specified matrix is positive definite.

**Definition:** check\_pos\_definite.hpp:29

Eigen.hpp

stan::math::check\_not\_nan

bool check\_not\_nan(const char \*function, const T\_y &y, const char \*name, T\_result \*result)

Checks if the variable y is nan.

**Definition:** check\_not\_nan.hpp:56


---

[ Stan Home Page ]

*© 2011–2014,
Stan Development Team.*
